# A Dataset of Univariate Crimp Force Curves for Data-Driven Time Series Analysis

**DOI:** 10.1038/s41597-025-05858-0

**Published:** 2025-08-26

**Authors:** Bernd Hofmann, Patrick Bründl, Jörg Franke

**Affiliations:** Friedrich-Alexander-Universität Erlangen-Nürnberg, Institute for Factory Automation and Production Systems (FAPS), Nuremberg, Germany

**Keywords:** Mechanical engineering, Electrical and electronic engineering

## Abstract

Data availability represents a critical bottleneck in the development of data-driven analysis tools, particularly for domain-specific applications in manufacturing. This paper introduces a comprehensive dataset of crimp force curves, captured during the production of crimp connections and commonly used for in-line quality control in industrial settings. The dataset comprises 2,439 crimp force curves, obtained from a semi-automatic crimping machine. Each curve has been annotated by both a state-of-the-art crimp force monitoring system, capable of performing binary anomaly detection, and by the authors who provided a more detailed classification into multiple quality categories. The paper introduces this novel dataset with the objective to enhance data-driven quality control systems in manufacturing. Specifically, the dataset serves two specific purposes: it provides a robust foundation for developing domain-specific machine learning models in the context of crimping processes, and it offers a benchmark resource for univariate time series analysis in data-driven applications.

## Background & Summary

**Fig. 1:**
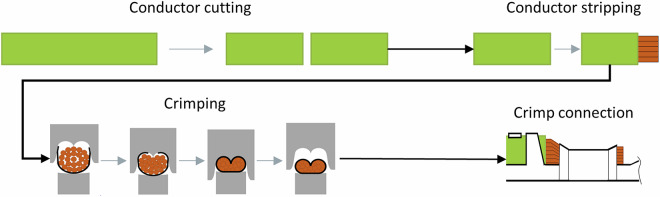
Schematic illustration of the crimping process, with conductor cutting and stripping as pre-processing steps.

Manufacturing companies continually face the challenge of finding the right balance between high-quality production and cost-efficient processes. In the context of crimping operations, implementing in-line quality control systems is paramount. Crimping is a solderless joining technique that creates permanent, electrically conductive and mechanically stable connections between conductors and terminals^1^. In serial production, the crimping process can be carried out manually using a stripping and crimping tool, semi-automatically with automated crimping, or fully automatically with pre-processing of the conductor included. Figure 1 illustrates the processing steps of crimping schematically.

In-line quality control systems typically employ crimp force curve monitoring, using manual feature extraction and predefined threshold settings to detect process deviations. Although recent research^2–4^ has demonstrated the significant potential of data-driven approaches in this domain, their adoption in real-world industrial settings remains limited. One key barrier to implementation is the discrepancy in data sovereignty between machine suppliers and manufacturers. Despite the fact that machine suppliers are well-positioned to develop and integrate monitoring tools for quality control or predictive maintenance, they lack access to the production data needed to create and validate data-driven solutions. In contrast, manufacturing companies do have access to the relevant data; however, they frequently lack the expertise, resources, or incentive to develop such tools themselves as this does not form part of their core business activities. In order to address this discrepancy, there is a significant need for open-access manufacturing datasets. The Crimp Force Curve Dataset^5^, which is the subject of this paper, was aggregated to support the advancement of data-driven methods for crimping processes. It provides a foundation for the development and evaluation of such approaches. Its effectiveness has been demonstrated in previous studies^4,6^, where subsets of the dataset were utilized to analyse crimp force curves from machines with different levels of automation and various wire cross-sections. The findings demonstrated that data-driven methodologies can effectively manage the variability intrinsic to crimping processes, exhibiting superior performance in comparison to conventional crimp force monitoring systems while maintaining transparency. The dataset consists of 2,439 annotated discrete crimp force curves, which were recorded during the operation of a semi-automated crimping machine. Each force curve has been assigned a label by both a state-o-the-art monitoring system and by the authors of this study. In contradistinction to the standard binary classification (OK/NOK) provided by the monitoring system, expert annotations offer a more nuanced labelling scheme with multiple quality classes. This facilitates the utilisation of the dataset for supervised multi-class classification tasks, enabling more effective fault detection compared to unsupervised anomaly detection methods. Within the domain of industrial quality control, the deployment of such algorithms provides operators with supplementary insights into particular fault types, thereby enhancing diagnostic precision and facilitating more efficient system troubleshooting. Beyond its utilisation for tasks such as anomaly detection and multi-class classification, it also serves as a foundational resource for training generalized machine learning models tailored to the crimping domain. Furthermore, the dataset is well-suited to the purpose of benchmarking univariate, discrete time series algorithms.

## Methods

### Experimental setup

**Fig. 2:**
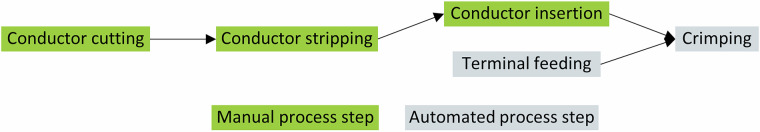
Flow chart of a semi-automatic crimping process.

All crimping operations were carried out using a semi-automatic crimping machine^7^, which automatically feds the terminals, while the conductors are manually inserted by the operator. The subsequent figure (Fig. 2) presents a schematic representation of a semi-automatic crimping process.

The crimping machine utilized in this study operates using an eccentric press and is capable of crimping wire-cross sections of up to 6 mm^2^ with a maximum force of 20 kN. Detailed machine specifications are provided in Table 1. The machine has been equipped with a force sensor^8^, which has been mounted directly onto the press’s connecting rod. The sensor continuously records the applied force during the crimping process, with each crimp connection resulting in one force curve being captured. This results in a univariate, discrete time series, in which the sensor signal varies over the press’s rotation angle, thereby effectively representing a force-over-displacement curve.

**Table 1:** Technical specification of the semi-automatic crimping machine.

Parameter	Specification
Machine model	Schaefer CrimpLine 240 ADVANCED (former: EPS2001)
Press drive mechanism	Eccentric
Terminal feeding	Mechanical
Pressing force	20 kN
Processable wire-cross section	up to 6 mm^2^
Stroke	40 mm
Closing height	135.78 mm
Weight	112 kg
Force sensor model	Engberts GmbH SDS100
Sensor type	Strain sensor

**Fig. 3:**
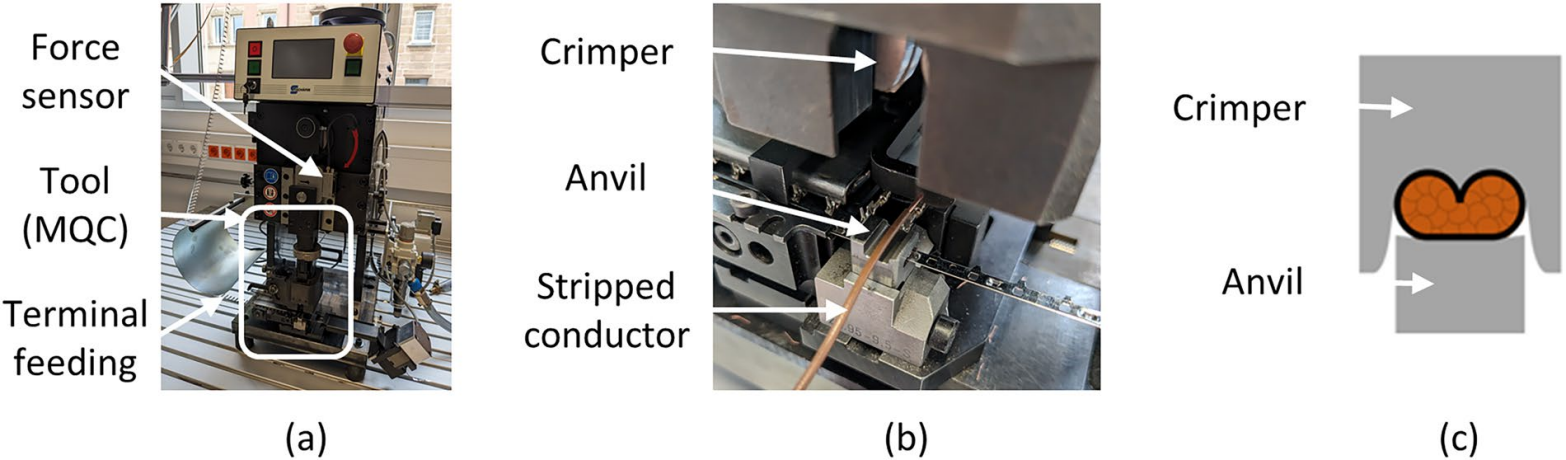
Experimental setup for data acquisition (**a**), close-up view of the MQC tool in operation (**b**), and schematic representation of the product-specific components and functional surfaces (**c**).

The Crimp Force Curve Dataset^5^ comprises two distinct conductor-terminal pairings: firstly, a 16-core, 0.50 mm^2^ FLRY-B copper conductor combined with an MLK 1.2 Sm Ag F-crimp connector, and secondly, a 12-core, 0.35 mm^2^ FLRY-B copper conductor paired with an MLK 1.2 ELA F-crimp terminal. Two modular quick-change (MQC) tools are utilised for the data acquisition, each configured for one of the specific conductor-terminal combinations. The standardised MQC system enables complete tool exchange when switching the product on a crimping machine. This approach ensures repeatable production conditions and prevents unintentional adjustments of functional components, thereby maintaining consistency in serial production and across experimental tests. These standardized tools differ only in two product-specific components, the crimper and the anvil, which are specifically tailored by the tooling manufacturer based on the geometry and type of the conductor and terminal. As illustrated in Fig. 3(c) only the crimper and anvil comprise functional surfaces and touch the material during operation.

**Fig. 4:**
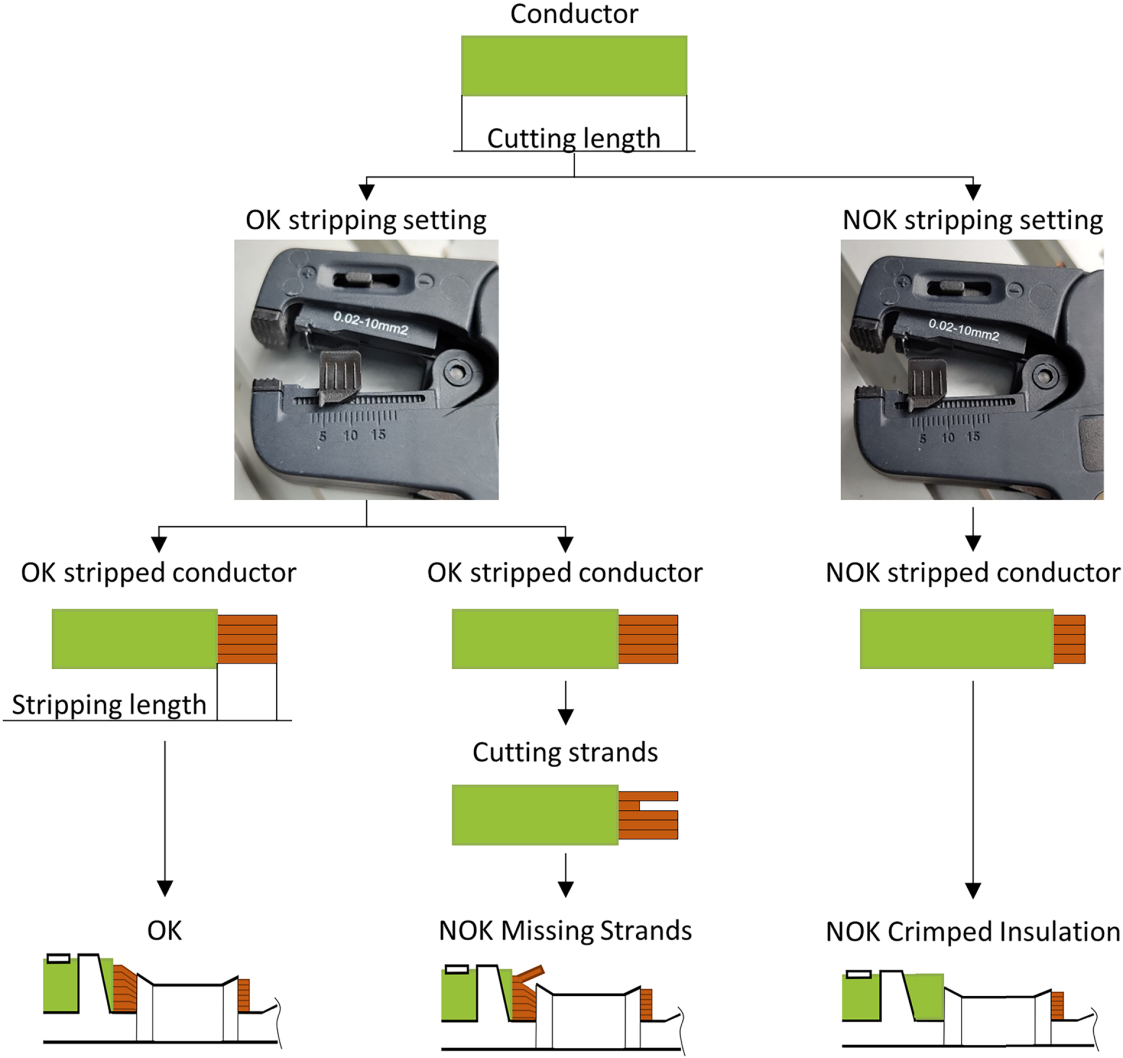
Manual preparation steps of the three quality classes in the semi-automatic workflow.

The crimping machine utilized in this study does not support conductor cutting or stripping operations, consequently, these preparatory steps were performed manually using a PHOENIX Contact WireFox 10^9^ stripping tool. This tool is capable of stripping conductors with a cross-section ranging from 0.02 to 10 mm^2^ and allows for a stripping length of up to 18 mm. Although the stripping length was precisely defined for the experiments, the conductor cutting length was not standardized, as it does not influence the crimping force curve. Consequently, minor variations caused by the manual operation in cutting length were tolerated and not documented. The integrated wire cutter of the stripping tool was used for the purpose of cutting. Conversely, a fixed stripping length was determined in order to differentiate between acceptable (OK) and defective (NOK) samples, given the significant effect that stripping length has on the crimp force curve. For the OK samples, the stripping length was set at 4 mm. For NOK samples of the Crimped Insulation class, a stripping length of 3 mm was utilized. Initial verification of stripping lengths was conducted through visual inspection by trained process experts, who evaluated the exposed conductor and the degree of insulation within the crimp. In addition to OK samples, two commonly observed defective categories were intentionally produced: Missing Strands and Crimped Insulation. The Missing Strands class was subdivided into three sub-classes, samples with one, two, or three strands removed. In order to create these, conductors were stripped to the OK length of 4 mm, after which the specified number of strands was manually removed. In the case of the Crimped Insulation class, the conductor was stripped to the NOK-defined 3 mm length, resulting in partial insulation being crimped. A comprehensive overview of the preparation procedures for all three quality classes is provided in Fig. 4.

### Data aggregation and annotation

The dataset is comprised of a series of smaller experiments conducted within the scope of the project entitled “Development of a machine learning based force curve analysis for a holistic process monitoring and quality assessment of crimp connections (DeepCrimpact)”. Despite the sequential execution of the recordings across subsequent sessions, all experiments were conducted under consistent laboratory conditions using material from the same batch and a standardized methodology, thereby ensuring the comparability of records. Data acquisition was carried out by various trained professionals, minimizing the likelihood of operator-related variability affecting the force curve measurements. The use of a standardized procedure further reduced the potential impact of external factors, such as the time of day. Additionally, a state-of-the-art crimp force monitoring system^10^ (BB07i) was incorporated into the experimental setup, which is capable of real-time detection of process deviations. Significant deviations, such as those caused by improper handling, would have been flagged as NOK, thereby providing an additional safeguard against external influences. In consideration of the aggregation of smaller series to one comprehensive dataset, it is important to note that the dataset was not intentionally designed to be balanced across the various quality classes. Instead, the objective was to generate a unified, representative dataset under stable process conditions. This approach enables users to implement task-specific balancing strategies that are tailored to their specific application contexts. The distribution of the class is outlined in Table 2.

**Table 2:** Class distribution of the Crimp Curve Dataset.

Wire-cross section	Quality class	Number of instances
0.5 mm^2^	OK	790
	One Missing Strand	76
	Two Missing Strands	188
	Three Missing Strands	51
	Crimped Insulation	117
0.35 mm^2^	OK	838
	One Missing Strand	96
	Two Missing Strands	103
	Three Missing Strands	0
	Crimped Insulation	180

**Fig. 5:**
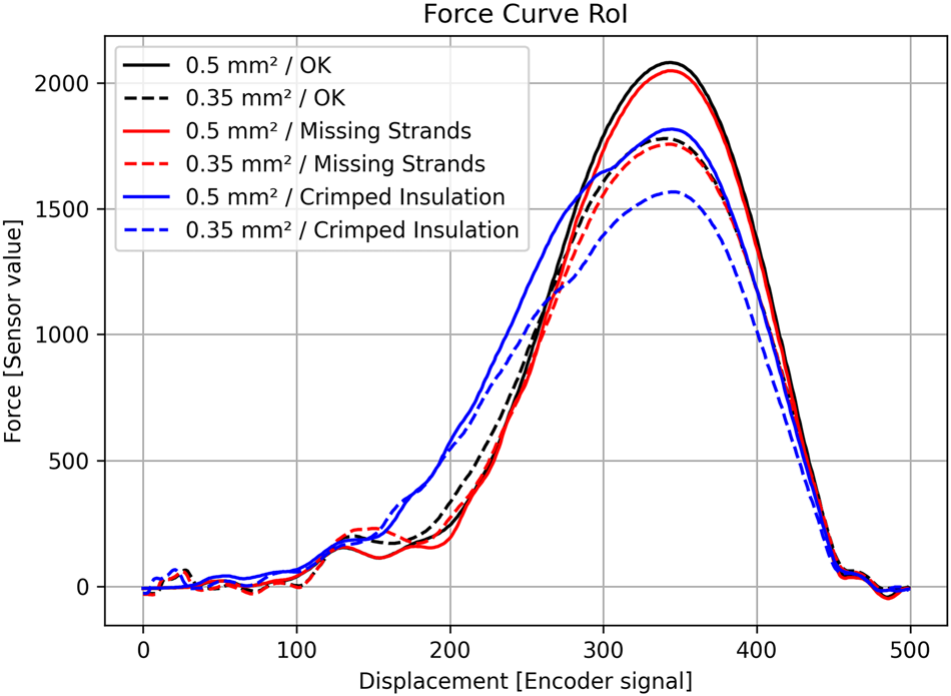
Exemplary instances of the three quality classes and their influence on the crimp force curve.

The dataset consists of a total of 2,439 crimp force curves, of which 1,222 have a wire cross-section of 0.50 mm^2^ and 1,217 have 0.35 mm^2^. 1,628 force curves can be assigned to the OK quality class, 297 to Crimped Insulation, 172 to the class with one missing strand, 291 to the class with two missing strands and 51 to the class of three missing strands. Figure 5 shows exemplary force curves of the different conductor samples and quality classes with their specific characteristics. It is evident that the 0.50 mm^2^ conductor requires a higher crimping force compared to the 0.35 mm^2^ conductor. Additionally, samples classified as Crimped Insulation exhibit a noticeably steeper force curve slope beginning at approximately data point 150, regardless of the conductor cross-section. In contrast, the Missing Strands defect class shows only a minor impact on the overall shape of the force curve, with deviations that are less pronounced.

### Data processing

**Fig. 6:**
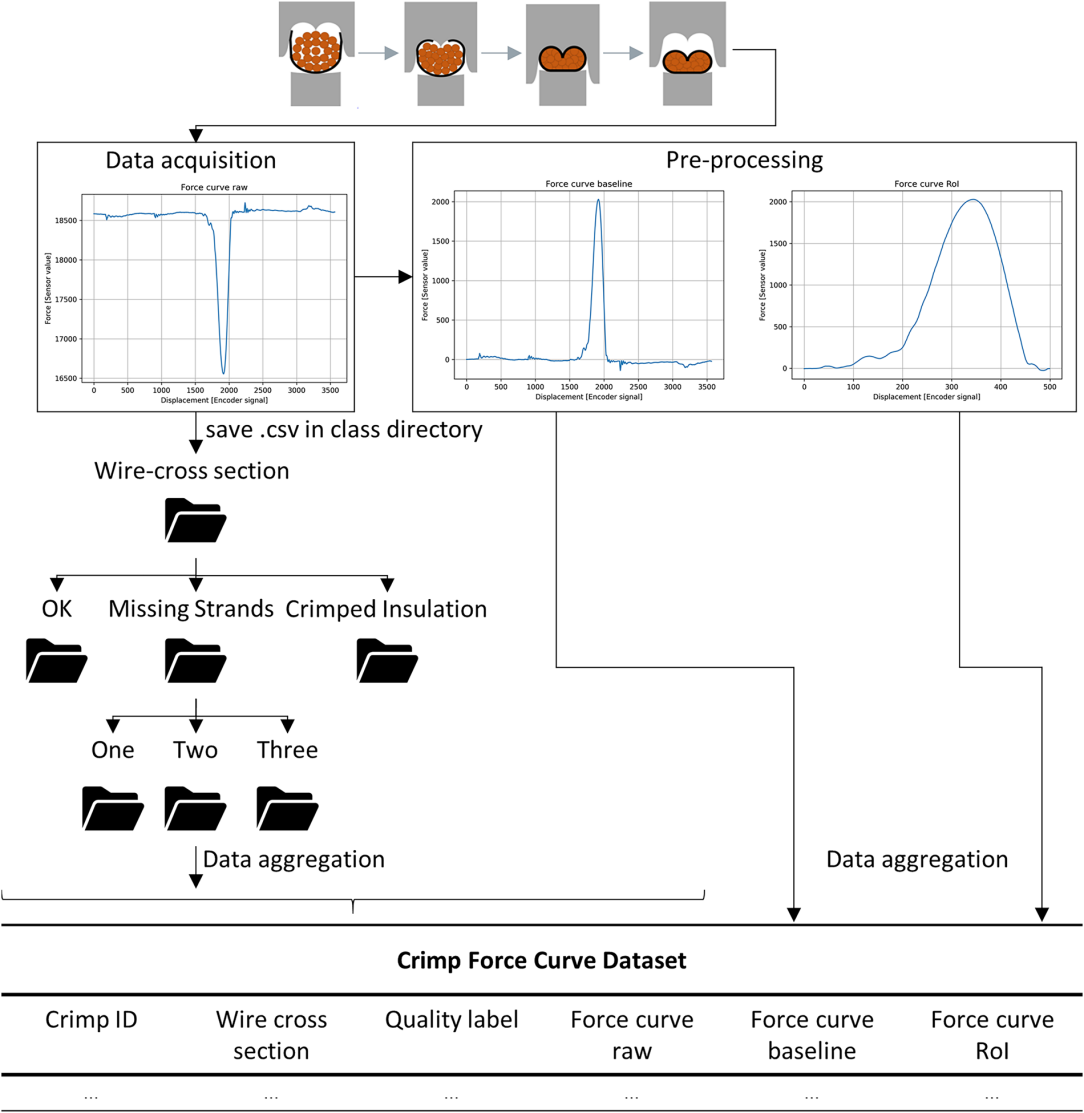
Data aggregation workflow.

Each recorded force curve was automatically saved as a CSV file and assigned a unique identifier (CrimpID) by the integrated crimp force monitoring system^10^. In addition to acquiring the raw force signal, labelled as “Force curve raw” and comprising of 3,566 data points, the system also undertook real-time anomaly detection by comparing the measured curve to a pre-recorded OK reference. Following the comparison process, the classification of each sample was automatically determined as either OK or NOK. The classification label was embedded directly within the corresponding CSV file alongside the CrimpID and force data. As previously delimitated, the conductor samples were prepared according to predefined quality classes. In order to ensure traceability and prevent cross-contamination between classes, a dedicated folder structure was implemented, with a separate directory assigned to each quality class. During the process of data acquisition, each quality batch of samples was recorded independently. For instance, 50 OK samples for the 0.50 mm^2^ conductor were initially recorded and stored in the designated OK directory. This process was repeated for subsequent batches, such as 100 Crimped Insulation samples, by manually switching to the appropriate folder prior to recording. The implementation of a structured directory system guaranteed the clear and systematic separation of the various quality classes. With the exception of the manual selection of the appropriate folder directory, it was not necessary to undertake any further annotation steps during the data collection process. The system automatically stored all relevant quality data, thereby negating the requirement for manual intervention in this regard. The data workflow is schematically illustrated in Fig. 6.

Subsequently, a total of 2,439 CSV files were retrieved from their designated metadata-specific directories. The files were loaded and aggregated into a single Pandas DataFrame, where each “Force curve raw” was stored as a NumPy array for subsequent processing. As a preliminary pre-processing step, each curve was inverted to account for the upside-down orientation of the integrated force sensor. Furthermore, a baseline correction was applied by subtracting the initial sensor reading from the entire curve. This ensured that all force curves began at a common reference level (y = 0), thereby enabling meaningful comparison across samples. The transformation applied to each raw force curve ${F}_{raw}=[{F}_{raw0},{F}_{raw1},\ldots ,{F}_{raw3565}]$ is defined by the following equation:

1 $${F}_{baseline}=-{F}_{raw}+{F}_{raw0}$$

However, only a specific segment, termed the region of interest (RoI), is typically retained for crimp force curve analysis. This RoI was defined in consultation with process experts from three different industrial partners and spans from data point 1575 to 2074. The extraction of the RoI from a baseline curve ${F}_{baseline}=[{F}_{baseline0},{F}_{baseline1},\ldots ,{F}_{baseline3565}]$ can be formally defined as:

2 $${F}_{RoI}=[{F}_{baseline1575},{F}_{baseline1576},\ldots ,{F}_{baseline2074}]$$

**Fig. 7:**
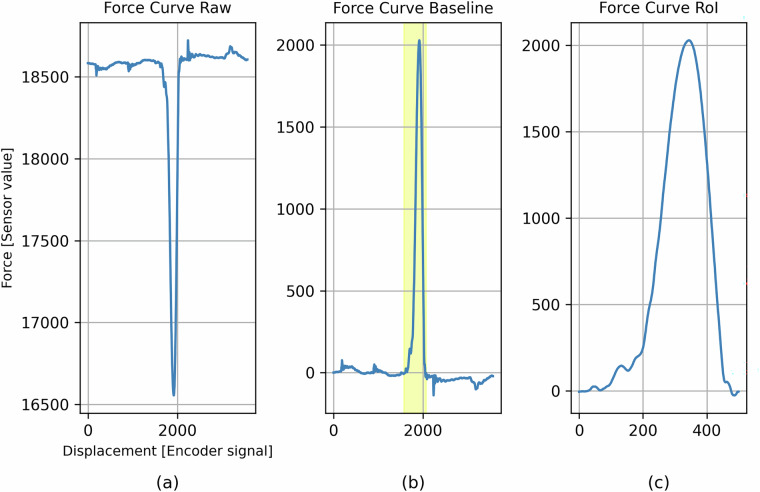
Processing steps from the raw curve (**a**) to the baseline (**b**) and the region of interest (**c**) of an exemplary instance.

The resulting RoI thus contains 500 data points and serves as the primary input feature for crimp force curve analysis. Despite the fact that the force sensor records additional data before and after this interval, these segments are not relevant for the evaluation of crimp quality, because the Crimper does not come into contact with the terminal before and after this interval. The definition of the relevant region and quality features is subject to variation between crimp force curve monitoring manufacturers. Therefore, the raw force curve is also provided in the dataset. The system utilised in this study, for instance, employs three distinct areas under the curve to differentiate between an OK or NOK connection. Figure 7 illustrates the transformation process from the raw sensor curve to the extracted region of interest.

## Data Records

The Crimp Force Curve Dataset^5^ is available at Harvard Dataverse. The annotated force curves are stored in a pickle file, which contains a pandas DataFrame. The DataFrame comprises 2,439 rows, which represent the instances, and includes a total of 11 columns, which are described as follows:

- CrimpID: Unique identifier assigned by the crimp force monitoring system as integer
- Wire_cross-section_conductor: Wire cross-section of the conductor as float [mm^2^]
- Force_curve_raw: Raw force curve with 3,566 datapoints as NumPy-array with integer values [Force sensor value]
- Force_curve_baseline: Baseline curve for comparability as NumPy-array with integer values [Force sensor value]
- Force_curve_RoI: Region of interest curve for crimp quality evaluation with 500 datapoints as NumPy-array with integer values [Force sensor value]
- Main_label_string: Quality class OK, Missing Strands or Crimped Insulation manually assigned by the authors based on the preparation steps as strings
- Main-label_encoded: Encoded quality classes OK, Missing Strands or Crimped Insulation to integers from 0 to 2
- Sub_label_string: Quality class OK, One Missing Strand, Two Missing Strands, Three Missing Strands or Crimped Insulation manually assigned by authors based on the preparation steps as strings
- Sub_label_encoded: Endocded quality classes OK, One Missing Strand, Two Missing Strands, Three Missing Strands or Crimped Insulation to integers from 0 to 4
- Binary_label_encoded: Binary encoded quality classes (OK, NOK) for anomaly detection manually assigned by authors to integers from 0 to 1
- CFM_label_encoded: Binary encoded quality classes (OK, NOK) assigned by the crimp force monitoring system to integers from 0 to 1

## Technical Validation

High data quality is essential for data-driven research, particularly in safety- and quality-critical manufacturing domains. The technical validation of the dataset is conducted across the following dimensions: completeness, accuracy, consistency, and uniqueness.

### Completeness

To evaluate completeness, the dataset was examined for missing data points and inconsistent curve lengths. The “Force_curve_raw” and “Force_curve_baseline” signals each consistently contain 3,566 data points, while the “Force_curve_RoI” signal contains 500 data points, indicating no variation in length. Annotation completeness was also verified, and no missing values have been found in any annotation columns. These findings confirm the dataset’s completeness.

### Accuracy

Accuracy is defined as the extent to which the dataset reflects the underlying real-world phenomena. Inconsistencies can impair machine learning models in their capacity to identify relevant statistical patterns and generalize effectively. The alignment with this aspect is supported by prior research^4^, which utilized a subset of the present dataset, augmented with data obtained from fully automated industrial manufacturing environments. The high performance achieved in that study underscores the consistency between the annotations of this dataset and those derived from real-world production settings, thereby reinforcing its accuracy. A secondary validation approach incorporated a comparison of the manual annotations to those generated by the integrated crimp force monitoring system. In 200 force curves (approximately 8% of the dataset) discrepancies in binary classification (OK/NOK) were observed. The instances are categorised as follows: 123 are labelled as “one missing strand,” 43 as “two missing strands,” 33 as “crimped insulation,” and one as “three missing strands.” It is important to note, that certain standards permit a maximum of 10% of strands to be absent^11^. Consequently, the crimp force monitoring system may not flag these cases as defects. Considering these standards for the 12- and 16-strand conductors utilised in the present dataset, annotation differences, especially for one to two missing strands, are deemed valid. Similarly, observed discrepancies within the Crimped Insulation class can be attributed to standard industrial tolerances. For instance, monitoring systems are only required to detect insulation material in the wire crimp above 30% of the total crimp length^11^. In this study, the insulation length for Crimped Insulation samples was consistently prepared at 3 mm. In contrast, the insulation length for the OK class was set at 4 mm, deliberately chosen as an edge case near the tolerance limit. However, due to manual insertion, slight variations in positioning are to be expected, which the monitoring system was not able to detect, because the 30% crimped insulation remained below the detection threshold. Importantly, no discrepancies were observed in the OK quality class, further supporting the overall accuracy and reliability of the dataset. Moreover, the implementation of a structured directory system during data collection ensured a clear and systematic separation of the various quality classes, thereby reinforcing the consistency of annotations across the dataset.

### Consistency

**Fig. 8:**
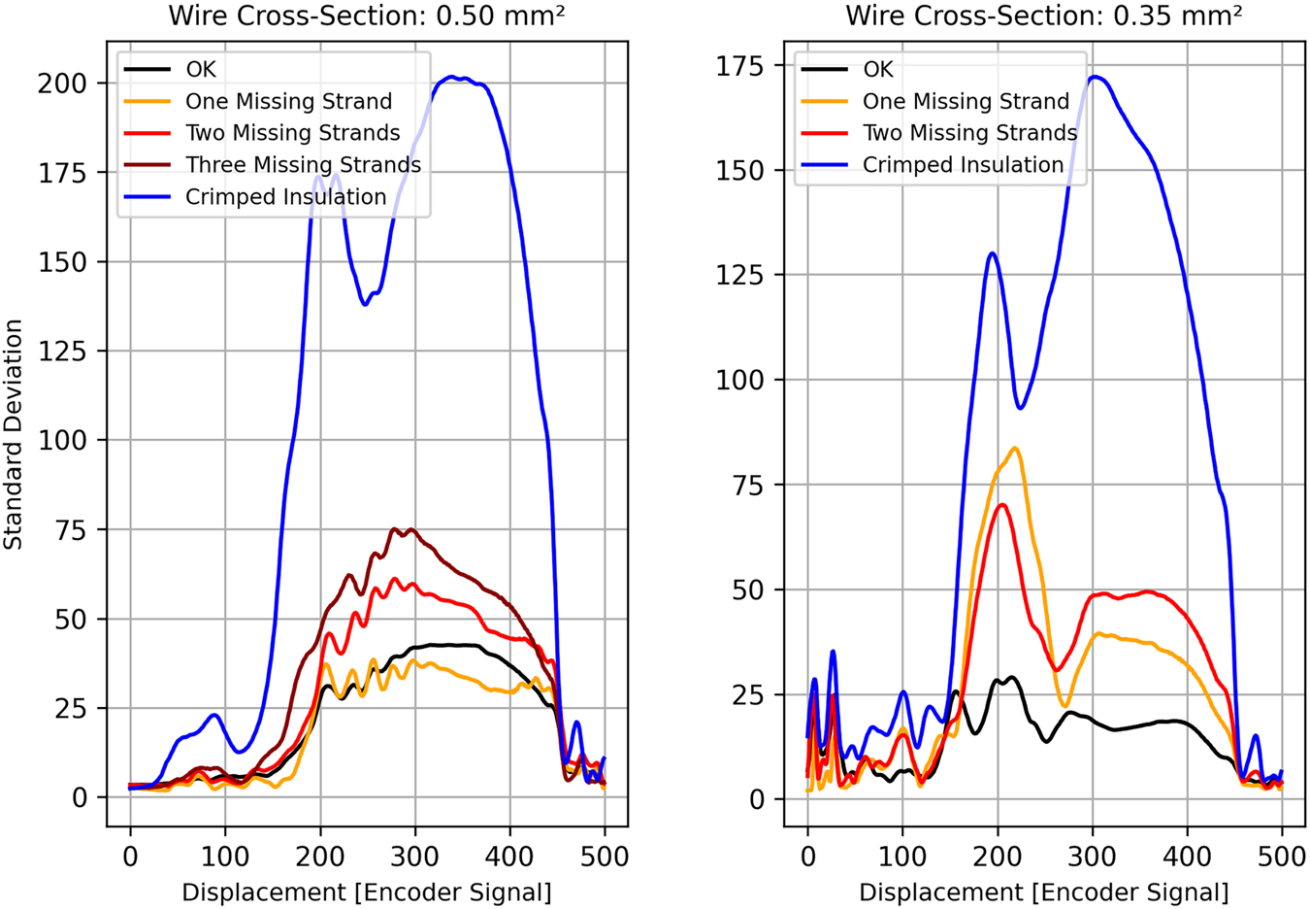
Consistency of the max datapoint of the three quality classes in the region of interest curve.

Consistency was assessed by analysing the standard deviation of each of the 500 datapoints within the RoI, separately for each quality class. In a consistent dataset, higher variability in the applied force is expected for more pronounced defect types, such as Crimped Insulation or Three Missing Strands. This is because, in the case of missing strands, individual strands have more freedom to move, leading to fluctuations in the force curve. Similarly, when insulation is crimped, the insulation material behaves inconsistently, which should be reflected in increased variability within the force curve. In contrast, lower variability is anticipated for the OK class or for samples with minor deviations, such as One or Two Missing Strands, which are still considered acceptable under certain industrial standards. Figure 8 illustrates the standard deviation profiles across all quality classes, separated by wire cross-section.

It is evident that the Crimped Insulation class consistently exhibits the highest variability among all quality classes for both the 0.50 mm^2^ and 0.35 mm^2^ conductors. Similarly, the Three Missing Strands class demonstrates above-average variability. The OK class exhibits the lowest variability for both conductor types, with One Missing Strand for the 0.50 mm^2^ conductor showing comparable or even lower variability in certain sections. This observation is plausible, as one missing strand in the 0.50 mm^2^ conductor represents approximately a 6% loss, which still within acceptable industrial tolerance limits. In contrast, the same defect has a more pronounced effect in the 0.35 mm^2^ conductor, where one missing strand already corresponds to over 8%, and two missing strands exceed 16%. Accordingly, increased variability is observed in these cases. It is also reasonable to observe differences in variability between the two conductor types, as Fig. 5 has demonstrated that their force curve characteristics differ substantially. Furthermore, the location of the missing strands within the conductor bundle can influence the shape of the force curve, since the spatial arrangement affects how the remaining strands behave during the compression phase. Overall, the observed variability across data points aligns with expectations for each quality class. These findings support the internal consistency and reliability of the dataset.

### Uniqueness

Uniqueness was verified using the automatically assigned crimp ID provided by the crimp force monitoring system. No duplicate IDs were found, confirming the uniqueness of all samples in the dataset.

## Usage Notes

The Crimp Force Curve Dataset is openly available at Harvard Dataverse 10.7910/DVN/WBDKN6. All processing and validation steps can be replicated using the code in https://github.com/BJhof/cfc-analysis.

### Experimental application

Parts of the dataset were already used in the field of machine learning-based fault detection and quality control of crimp force curves^4,6^. In these proposed concepts meaningful fault detection scores were achieved across various conductor-terminal pairings.

## Data Availability

To ensure reproducibility, all software used for processing and validating the force curves, as well as for generating the plots shown in Figs. 5, 7, 8, is openly available at https://github.com/BJhof/cfc-analysis. The analysis was conducted using open-source Python libraries, including NumPy^12^, Pandas^13^ and matplotlib^14^.
